# Comprehensive insights on genetic alterations and immunotherapy prognosis in Chinese melanoma patients

**DOI:** 10.1038/s41598-024-65065-6

**Published:** 2024-07-18

**Authors:** Dong-Dong Jia, Tao Li

**Affiliations:** Department of Bone and Soft-Tissue Surgery, Zhejiang Cancer Hospital, Chinese Academy of Sciences, Hangzhou Institute of Medicine (HIM), No.1 Banshan East Road, Hangzhou, 310022 Zhejiang China

**Keywords:** Melanoma, Genetic profiling, Immune checkpoint inhibitors, Prognostic markers, ALK expression, Melanoma, Cancer genomics, Cancer

## Abstract

Immune checkpoint inhibitors (ICI) have emerged as a promising therapeutic option for melanoma, which demonstrating improved clinical outcomes in melanoma patients regardless of specific genetic mutations. However, the identification of reliable biomarkers for predicting immunotherapy response and prognosis remains a challenge. In this study, we performed genetic profiling of the melanoma patients with different subtypes and evaluated the efficacy of ICI treatment. A total of 221 melanoma patients were included in our cohort, consisting primarily of acral lentiginous melanoma (ALM), cutaneous malignant melanoma (CMM), and mucosal malignant melanoma (MMM). Genetic analysis revealed *BRAF* mutations was predominant in CMM and *NRAS* mutations was prevalent in ALM. Copy number variants (CNVs) and structural variants (SV) were also detected, with *CCND1* and *CDK4* being the most affected genes in CNV and *BRAF, ALK* and *RAF1* being the druggable targets in SV. Furthermore, *NRAS* mutations were associated with a poor prognosis in ALM, while *TERT* mutations were linked to unfavorable outcomes in CMM after receiving PD-1 therapy. Additionally, *ALK* expression exhibited improved outcomes in both ALM and CMM subtypes. Our study provides a comprehensive genomic and pathological profiling of Chinese melanoma patients, shedding light on the molecular landscape of the disease. Furthermore, numbers of gene mutations and ALK expression were identified as prognostic indicators. These findings contribute to the understanding of melanoma genetics in the Chinese population and have implications for personalized treatment approaches.

## Background

Melanoma, despite significant advancements in its management over the past decade, remains the most fatal form of skin cancer^1^. Incidence rates and outcomes vary across different geographical regions, with Australia, New Zealand, Northern Europe, and North America reporting the highest rates^2^. According to a report from the International Agency for Research on Cancer, in 2020, over 320,000 new cases of melanoma were diagnosed, resulting in more than 57,000 deaths. Furthermore, the number of new cases of cutaneous melanoma (CMM) per year will increase by more than 50% from 2020 to 2040^3^. CMM is the predominant histological subtype in Caucasians, often detected early due to its visible location. However, Asian patients with low incidence rate commonly present with acral lentiginous melanoma (ALM), which leads to delayed diagnoses and poorer prognosis^4^.

Thanks to collaborative work that unveiled the role of oncogenic driver mutations in melanoma. Significant efforts have been made to explore potential treatment methods, and strategies have substantially improved. Numerous oncogenic drivers, including but not limited to *BRAF, NRAS* and *TERT*, have been identified in melanoma^5–7^. Among them, *BRAF V600E/K* stands out as the most effective target and has become the only target in melanoma targeted therapies approved by the food and drug administration^8^. Numerous studies have demonstrated that dabrafenib and trametinib combined therapy are the best options for melanoma patients with *BRAF* mutations, and superior outcomes were verified in multiple clinical trials^9–11^. However, while the high frequency of *BRAF* mutations (20–30%) and excellent objective response rate (ORR 60–70%) indicate the clinical value of *BRAF*-targeted therapy, the remaining 80% of melanoma patients do not benefit from targeted therapy.

In contrast to targeted therapy for *BRAF V600E/K* mutant melanomas, immune checkpoint inhibitors (ICI) have shown enhanced clinical outcomes in melanoma patients irrespective of the presence of a particular oncogenic mutational signature or biomarker^12,13^. Previous studies demonstrated that 40–60% melanoma patients have shown a positive response to ICI^14,15^. These findings collectively illustrate the potential of ICI therapy to benefit a broader range of melanoma patients and extend patient lifespans.

Several gene mutations have been identified as potential biomarkers for predicting the response to immunotherapy^16–18^. As mentioned above, one of the most extensively studied mutations is the *BRAF* mutation, which is present in notable proportion of melanoma cases. Melanoma patients with *BRAF* mutations have shown favorable responses to ICIs. Additionally, *NRAS* mutations are better prognosis indicators in melanoma patients received ICIs^19^. Therefore, it is imperative to conduct research endeavors focused on comprehending the clinical implications of vital molecular alterations and identify predictive and prognostic markers for treatment response. Such efforts are vital for improving the treatment outcomes of ICIs therapy.

This study aimed to investigate the molecular characteristics of Chinese melanoma patients across various histological subtypes. The genetic landscape of the patients was also profiled, and the efficacy of ICIs treatment was evaluated. Additionally, the study uncovered the association between genetic alterations and prognostic outcomes.

## Methods

### Patients population

Tumor histological specimens (formalin-fixed paraffin-embedded tissues or frozen tissues) obtained Cancer Hospital of the University of Chinese Academy of Sciences (Zhejiang Cancer Hospital) between March 2018 and May 2022 were used for NGS detection. All patients were diagnosed via pathology, imaging, and clinical findings. Clinical data were obtained from the patients’ records. The study was conducted in accordance with The Code of Ethics of the World Medical Association (Declaration of Helsinki) for experiments involving humans and within guidelines and regulations by the Research Ethics Committee of the Zhejiang Cancer Hospital.

### Pathological diagnosis

The pathological features of patients were identified based on the NCCN guideline criteria^20^. ALM, CMM and MMM were diagnosed based on the site of disease. ALM appears on the palms of the hands, soles of the feet, or under the nails. CMM refers to melanoma that occurs on the skin’s surface. It often develops in areas exposed to the sun, such as the face, arms, legs, and back. MMM occurs in the mucous membranes lining areas like the mouth, nose, throat, vagina, or anus.

### NGS assay

Between 10 and 500 ng of TNA was used as an input template for NGS library construction beginning with an RT reaction that converted RNA to cDNA without altering carryover gDNA. Input template was mixed with random hexamers and heated to 65 ℃ for 5 min, and immediately chilled in icy water. Reverse transcriptase,RNase inhibitor, and dNTPs (Enzymatics) were added and incubated at 42 ℃ for 2 h, followed by 70 ℃ for 15 min. The second strand was synthesized with Escherichia coli DNA polymerase I and RNase H (Enzymatics) at 16 ℃ for 1 h. After SPRI beads cleanup (Beckman), DNA were enzymatically fragmented by DNA fragmentation enzyme mix (New England Biolabs or Kapa Biosystems) and end-polished by T4 DNA polymerase, Taq polymerase, and T4 polynucleotide kinase (Enzymatics). This polished fragment is ligated with a set of adaptors. Each adaptor consisted of a designated P5 index for sample deconvolution and a degenerate unique molecular identifier (UMI) for amplicon binning. Ligated templates were SPRI-cleaned and mixed with the first pool of target-specific primers and thermal stable DNA polymerase (Thermo Fisher), and subjected to multiple linear amplification cycles, each producing a complementary strand copy of the input template. All primers share a common 5 tag suppressing amplification between genespecific primers owing to hairpin formation in such amplicons. After cleanup, a second pool of target-specific primers nested to the first pool, a common adaptor primer, and an indexed P7 primer were applied for PCR amplification. Resulting libraries were pooled and sequenced using HiSeq 10 or NextSeq500/550 sequencers (Illumina) with paired-end 150 cycles and 8-bit P7 index run setting. A set of 96 P5 adaptor indexes and matched P7 primer indexes were designed, allowing library pooling for sequencing.

### NGS data processing

Raw data were demultiplexed using Illumina bcl2fq version 2.19 for P7 index, followed by a custom script for matched P5 index demultiplexing, BBduk for adaptor trimming, and UMI parsing. Fastq sequences were aligned to human reference genome (hg19) using BWA MEM with the default setting. On-target alignments were extracted using BedTools 2.27^21^ supplied with specific panel bed files. SNVs were called using a UMI-aware custom script that includes samtools and bcftools. SNVs with occurrence frequency higher than 1% in the dbSNP database were regarded as polymorphisms and filtered. Gene fusions were called based on BWA MEM supplementary alignments, taking into account different mapping start positions, and the breakpoint frame status was inferred based on RefSeq open reading frames. We favored BWA MEM over common RNA-seq aligners such as STAR, TOPHAT, or Mapsplice^22^ because its algorithm was advantageous for fusions involving 2 different genes. BWA MEM also allowed for simultaneous gDNA (resulting bam file is compatible with the mutation calling module) and cDNA read alignment, which was unique to our wet laboratory method.

### Statistical analysis

Survival data were analyzed with the Kaplan–Meier method, and log-rank tests were used for comparisons between different groups. Cox proportional hazards regression analysis was conducted to identify the following possible predictors of RFS in melanoma patients: *NRAS* status (positive vs negative), *TERT* status (positive vs negative), *ALK* mRNA status (positive vs negative), *BRAF* status (positive vs negative), *KIT* status (positive vs negative), KRAS status (positive vs negative) and TP53 status (positive vs negative). All statistical analyses were conducted using GraphPad Prism (version 8.02) software. The results are expressed as the mean ± SEM. The data were analyzed using a t test or one-way ANOVA, followed by the appropriate post-hoc tests (GraphPad Prism 7, GraphPad, CA, USA).

### Ethical approval and consent to participate

The study was approved by Ethics Committee of the Zhejiang Cancer Hospital (IRB-2021-301), and informed consent was obtained from all subjects.

## Results

### Patient characteristics and genetic alterations

A total of 221 melanoma patients were included in the cohort (Table 1). A significant difference was found in genders, with the number of males being twice as high as females (Table 1, 63% vs 37%, P < 0.05). The age distribution ranged from 20 to 90 years, with an average age of 62 years for the cohort.

**Table 1:** Clinicopathologic features of melanoma patients.

Clinicopathologic features (n = 221)	
Age	
Average	61.4 years
Median	62 years
Range	20–90 years
Sex	
M	139 (63%)
F	82 (37%)
Pathological types	
Acral lentiginous melanoma	126 (57.0%)
Feet	109 (86.5%)
Hands	16 (12.7%)
Other	1 (0.8%)
Cutaneous melanoma	81 (36.7%)
Leg	22 (27.1%)
Abdomen	16 (19.7%)
Back	12 (14.8%)
Head and neck	11 (13.5%)
Chest	5 (6.1%)
Arm	4 (4.9%)
Others	11 (13.5%)
Mucosal melanoma	14 (6.3%)
Head and neck	8 (57.1%)
Colon	2 (14.2%)
Genitals	2 (14.2%)
Vulva	2 (14.2%)

**Figure 1:**
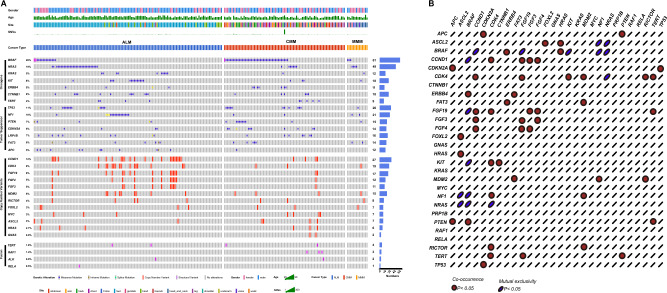
The characteristics of the gene mutations in melanoma patients identified by DNA sequencing. (**A**) the spectrum of the variations (SNV, CNV and fusion) in the ALM, CMM and MMM; (**B**) the distribution of gene mutations based on mutation types; (**C**) The co-occurrence and mutual exclusivity of top genes detected in the melanoma. *SNV* single nucleotide variant, *CNV* copy number variant, *ALM* acral lentiginous melanoma, *CMM* cutaneous malignant melanoma, *MMM* mucosal malignant melanoma.

Furthermore, comprehensive sequencing was performed on 221 tissues from melanoma patients to profile the genetic landscape (Fig. 1A). The mutated genes were categorized based on the type of mutation: single nucleotide variant (SNV), copy number variant (CNV), and fusion variant (SV). Various pathways were involved in the cohort, with more than half of the SNVs occurring in genes (*BRAF, NRAS*, and *KRAS*) in the MAPK pathway (n = 119, n = 53%). Specifically, *BRAF, NRAS,* and *KRAS* accounted for 26% (n = 58), 22% (n = 49), and 5% (n = 12) of the mutations, respectively. Among the *BRAF* mutations, *BRAF V600E* (n = 45, 77%) and *BRAF V600K* (n = 5, 9%) were the predominant variants. Additionally, *BRAF D22N, BRAF D594A, BRAF D594N, BRAF I554T, BRAF N581S*, and *BRAF S467L* were observed once in the study. *NRAS* SNVs were mainly found in *G61* (n = 23, 56%), *G12* (n = 9, 22%), and *G13* (n = 9, 22%). A similar pattern was observed for KRAS. Furthermore, two receptor tyrosine kinase genes, *KIT* (n = 18, 8%) and *ERBB2* (n = 11, 5%), were also detected. Several tumor suppressor genes with a significant proportion were identified in the cohort, such as *TP53* (n = 26, 11%) and *NF1* (n = 21, 10%).

The average number of CNV was assessed in the cohort, and an average of 5.411 alterations were identified. The most affected gene was *CCND1* (n = 27, 12%), which is involved in cell cycle control. *CDK4*, a receptor tyrosine kinase, was also detected in 8% of patients. *FGF19* (n = 17, 8%) was the third most affected gene, followed by *FGF3* and *FGF4*, both belonging to the fibroblast growth factor family. Furthermore, gene mutations in the *RP-MDM2-P53* pathway, including *MYC* and *MDM2*, also exhibited high frequency. In the SV group, five drivers were identified, with *TERT* being the most common (n = 4, 1.8%), followed by *BRAF* (n = 3, 1.4%), *RAF1* (n = 3, 1.4%), *ALK* (n = 2, 0.9%), and *RELA* (n = 1, 0.5%).

To evaluate the association between genes, co-occurrence and mutual exclusivity analyses were conducted for the highly frequent genes (Fig. 1B). CNVs showed a high frequency of co-occurrence (Fig. 1B). For example, *CCND1* had a significant co-occurrence with *CDK4, FGF3/4/19* (P < 0.05). Additionally, sporadic SNVs were also found in co-occurring genes. Co-occurrence was observed between *BRAF* and *ERBB4, PTEN*. Mutual exclusivity was mainly observed with *BRAF*, for instance, it was mutually exclusive with *CCND1, FGF19, KIT, NF1,* and *NRAS*.

### Clinicopathologic and molecular features difference between melanoma subtypes

**Figure 2:**
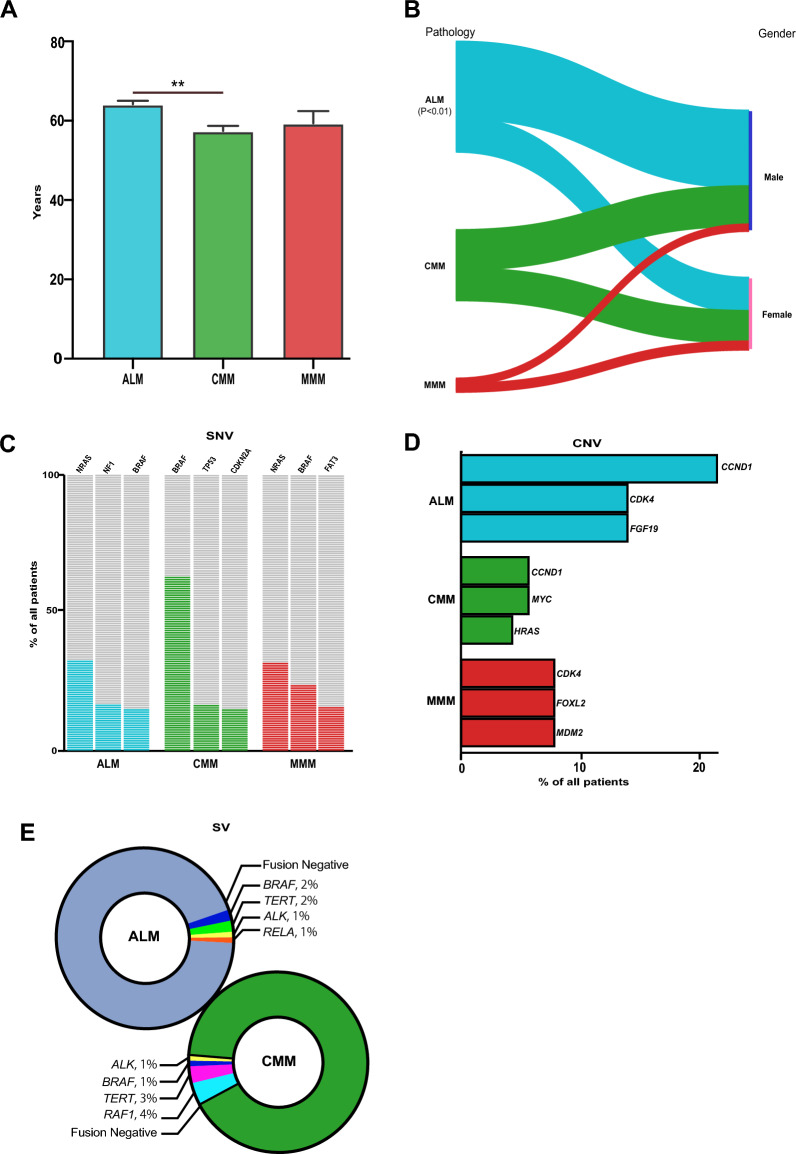
The features of clinical characters and gene distributions between melanoma subtypes. (**A**) the age distribution between subtypes. The age distribution in ALM was significantly higher than CMM (P < 0.01); (**B**) the gender comparison between subtypes. In the ALM, the number of male was significantly higher than female (P < 0.01); (**C**) the prevalence of gene mutants between subtypes; (**D**) the top 3 genes in SNV between subtypes. *NRAS*, *NF1* and *BRAF* were the top3 genes in ALM. *BRAF, TP53* and *CDKN2A* were the top3 genes in CMM. *NRAS, BRAF* and *FAT3* were the top3 genes in MMM; (**E**) the top 3 genes in CNV between subtypes. *CCND1, CDK4* and *FGF19* were the top3 genes in ALM. *CCND1, MYC* and *HRAS* were the top3 genes in CMM. *CDK4, FOXL2* and *MDM2* were the top3 genes in MMM; (**F**) the fusion drivers in ALM and CMM. **P < 0.01. *SNV* single nucleotide variant, *CNV* copy number variant, *ALM* acral lentiginous melanoma, *CMM* cutaneous malignant melanoma, *MMM* mucosal malignant melanoma.

The pathologic diagnosis was conducted and 3 different pathological subtypes were identified (Table 1), including ALM (n = 126, 57%), CMM (n = 81, 36.7%) and MMM (n = 14, 6.3%). Obviously, ALM accounted a significant proportion in the cohort, which was 9 times higher than MMM. The location of symptoms was diverse in each subtype (Table 1). In the ALM group, feet (n = 109, 86.5%) was the dominate site, followed by hands (n = 16, 12.7%). Compared to the ALM, the site of onset was more diverse in CMM. Leg (n = 22, 27%) was the primary site in CMM. Abdomen (n = 16, 20%) and back (n = 12, 15%) were found to be the second and third location. In the MMM group, head and neck was the frequent site at diagnosis. In order to describe the clinicopathologic features between subtypes. The age at diagnosis was analyzed and the significant difference was found between ALM and CMM, which demonstrated the higher age in ALM group (Fig. 2A, P < 0.01). Moreover, a significant difference in gender distribution was found in ALM, the number of male (n = 89) was much higher than female (n = 39) (Fig. 2B, P < 0.01).

In order to identify genetic distribution difference between melanoma subtypes, the sequencing results were further analyzed based on subtypes. The number of high frequent genes was similar between ALM (n = 28) and CMM (n = 29), which was twice higher than MMM (n = 13). Although the gene number was similar between ALM and CMM, the genetic patterns were different (Fig. 2C). The top 3 genes with SNVs in ALM were *NRAS* (n = 34, 31%), *NF1* (n = 17, 16%) and *BRAF* (n = 15, 14%). In the CMM group, *BRAF* (n = 43, 61%) was the most frequent gene, which was much higher than *TP53* (n = 11, 15%) and *CDKN2A* (n = 10, 14%). The genetic distribution pattern in MMM was similar to ALM, *NRAS* (n = 4, 31%) carried the highest proportion, followed by *BRAF* (n = 3, 23%) and *FAT3* (n = 2, 15%). Different from SNV, the proportion of top3 genes with CNVs was similar. *CCND1* was the primary gene in both ALM and CMM, followed by *CDK4* and *FGF19* in ALM, *MYC* and *HRAS* in CMM. In MMM, *CDK4, FOXL2* and *MDM2* shared same proportion (Fig. 2D). The number of oncogenic driver genes in fusions was less than SNV and CNV (Fig. 2E). Totally, only 5 driver genes were identified in the cohort. ALM and CMM carried 4 of 5, respectively. In ALM, *BRAF* and *TERT* had the highest proportion followed by *ALK* and *RELA*. Similarly, besides *RAF1, TERT, BRAF* and *ALK* were also found in CMM. There was no fusions in MMM.

### Characteristics of patients treated with immune checkpoint inhibitors

**Figure 3:**
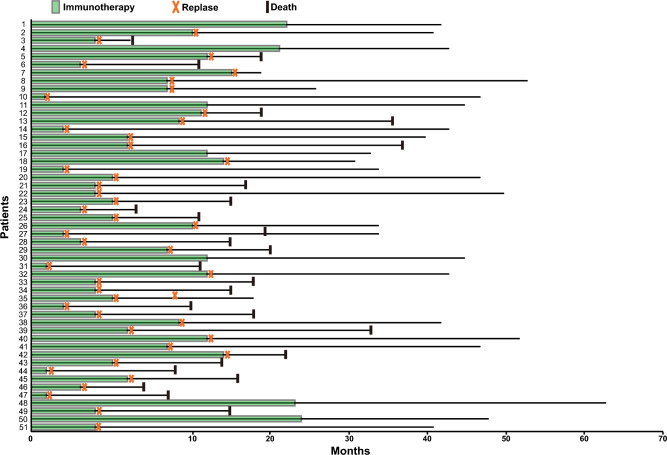
The monitoring results of PD-1 therapy in 51 patients.

In total, 51 patients in the cohort received ICIs treatment (Fig. 3). The number of patients with ALK, CMM and MMM were 33, 17 and 1, respectively. Seven patients did not reach disease progress from ICIs treatment still now. The average length of relapse free survival (RFS) of those 7 patients was 41.2 months. Among them, the longest RFS was 53.6 months, followed by 48.2 months, 45.2 months, 43.8 months, 42.3 months, 33.5 months and 21.9 months. In the patients who were found to be disease progress (n = 44), the RFS length was variable, which was between 0.7 years to 35.6 months. Among them, the RFS of 38 patients were less than 12 months.

### Clinical outcomes of PD-1 therapy in patients with different gene mutations

**Figure 4:**
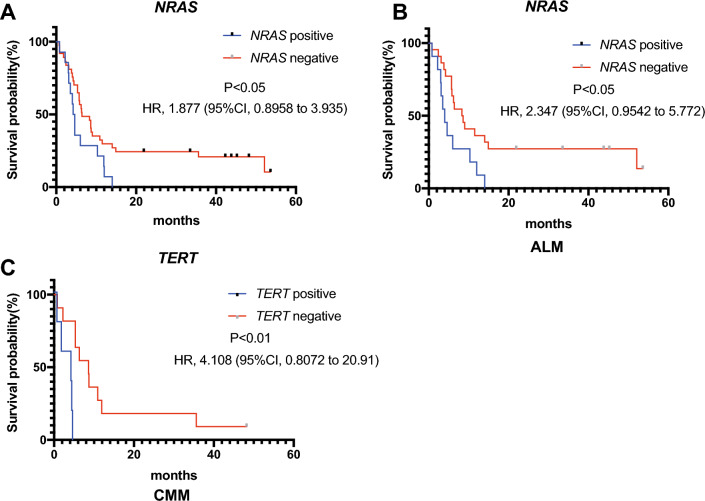
The Kaplan–Meier curves for RFS in patients with SNVs after PD-1 therapy in melanoma. (**A**) *NRAS* mutation is the negative prognostic indicator of PD-1 therapy in melanoma without distinguishing pathological subtypes. (**B**) *NRAS* mutation is the negative prognostic indicator of PD-1 therapy in ALM. (**C**) *TERT* mutation is the negative prognostic indicator of PD-1 therapy in CMM. *SNV* single nucleotide variant, *ALM* acral lentiginous melanoma, *CMM* cutaneous malignant melanoma. Positive, the specific gene mutation (*NRAS* or *TERT*) was detected in patients by DNA sequencing. Negative, the specific gene mutation (*NRAS* or *TERT*) was not detected in patients by DNA sequencing. *RFS* relapse-free survival.

The association between gene mutations and prognostic outcomes were further analyzed. Firstly, the highly frequent genes in the cohort were included in the analysis. There was no significant difference in prognostic outcomes between *BRAF, KIT, KRAS* and *TP53* mutant and wild groups (Fig. S1A–D). On the contrary, *NRAS* was found to be highly related to poor prognosis (Fig. 4A, HR, 1.877, 95% CI 0.8958 to 3.935; P < 0.05). Furthermore, the effect of clinicopathologic subtypes on prognostic outcomes was further explored. However, there was no significant difference was found between ALM and CMM (Fig. S1E). Interestingly, the particular gene mutation in ALM and CMM was significantly related to the prognosis. In the ALM, the RFS for *NRAS* mutant was significant lower than wild type group (Fig. 4B, HR 2.347, 95% CI 0.9542 to 5.772; P < 0.05). CMM displayed a different pattern, the significant difference in prognosis was found in *TERT* (Fig. 4C, HR 4.108, 95% CI 0.8072 to 20.91; P < 0.01).

**Figure 5:**
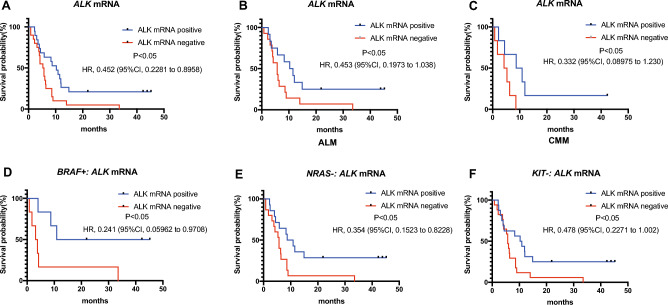
The Kaplan–Meier curves for RFS in patients with *ALK* mRNA after PD-1 therapy in melanoma. (**A**) *ALK* mRNA expression is a positive prognostic indicator in melanoma without distinguishing pathological subtypes. (**B**) *ALK* mRNA expression is a positive prognostic indicator in ALM. (**C**) *ALK* mRNA expression is a positive prognostic indicator in CMM. (**D**) *ALK* mRNA expression could positively impact the prognostic outcomes of patients with *BRAF* mutation. (**E**) *ALK* mRNA expression could positively impact the prognostic outcomes of patients without *NRAS* mutation. (**F**) *ALK* mRNA expression could positively impact the prognostic outcomes of patients without *KIT* mutation. *ALM* acral lentiginous melanoma, *CMM* cutaneous malignant melanoma. Positive, the *ALK* mRNA was detected in patients by RNA sequencing. Negative, the *ALK* mRNA was not detected in patients by RNA sequencing. *RFS* relapse-free survival.

Additionally, the *ALK* mRNA level was found to be closed associated to the prognostic outcomes. Patients with positive expression of *ALK* had longer RFS than those without *ALK* mRNA (Fig. 5A, HR 0.452, 95% CI 0.2281 to 0.8958; P < 0.05). Furthermore, *ALK* mRNA displayed positive effect on RFS in both ALM (Fig. 5B, HR 0.453, 95% CI 0.1973 to 1.038; P < 0.05) and CMM (Fig. 5C, HR 0.332, 95% CI 0.08975 to 1.23; P < 0.05). Moreover, *ALK* mRNA exhibited different effect on patients with SNVs. *ALK* mRNA could positively impacted the prognosis of patients with *BRAF* mutant (Fig. 5D, HR 0.241, 95% CI 0.05962 to 0.9708; P < 0.05), *NRAS* wild (Fig. 5E, HR 0.354, 95% CI 0.1523 to 0.8228; P < 0.05), and *KIT* wild (Fig. 5F, HR 0.478, 95% CI 0.2271 to 1.002; P < 0.05) groups.

## Discussion

Melanoma is renowned for its extensive genetic heterogeneity, with various genetic mutations driving its initiation, progression, and resistance to therapy^23^. The identification and understanding of these genetic mutations hold significant importance for subsequent therapeutic interventions, including immunotherapy. In this study, we aimed to comprehensively profile the genetic landscape of Chinese melanoma patients with different subtypes. Our analysis revealed numerous oncogenes and tumor suppressors affected by SNVs, such as *BRAF, NRAS,* and *NF1*. Notably, the *BRAF V600E* mutation accounted for a substantial proportion of *BRAF* variations. Furthermore, we identified *CCND1, CDK4,* and *FGF9* as the top three genes with the highest frequency of CNVs. Additionally, we discovered several oncogenic drivers associated with rearrangements, including *BRAF, RAF1,* and *ALK*. In addition to characterizing the genetic landscape, we explored the association between genetic mutations and the outcomes of immunotherapy. Our findings indicated a negative prognosis associated with *NRAS* mutations in ALM. Similarly, we observed unfavorable prognostic outcomes in CMM patients with *TERT* mutations. Moreover, we identified a relationship between *ALK* mRNA levels and immunotherapy efficacy, as patients with *ALK* expression exhibited improved prognostic outcomes in both ALM and CMM.

In contrast to the high incidence of Caucasian melanoma, melanoma morbidity is lower in the Asian population^24,25^. This disparity in incidence highlights the extensive findings that can be drawn from melanoma studies conducted on Caucasian populations. However, research on melanoma in the Asian population remains limited due to its lower occurrence. Previous studies have shown that CMM (> 90%) is more common among Caucasian populations, particularly those with fair skin and a high susceptibility to ultraviolet (UV) radiation^26^. On the contrary, ALM is prevalent among Asian populations, as reported in previous Asian studies^27^. In our cohort, ALM accounted for the majority (57.4%), followed by CMM (36%) and MMM (6.6%). In terms of somatic gene mutations, the mutation rates of *BRAF, NRAS, NF1, KIT,* and *TERT* were 26, 19, 10, 8, and 4%, respectively. The mutation rates of *BRAF, NF1*, and *TERT* were like those reported in previous studies on Asian populations^28^. However, the *NRAS* mutations in our cohort was almost twice higher than the proportion in other studies (19% vs 10%). This difference may be attributed to the significant number of *NRAS*-mutant patients in our specific cohort or the prevalence of synonymous mutations in this cohort. In both our cohort and other studies^28,29^, *BRAF* mutations were predominantly *V600* hotspot mutations, particularly *V600E*, which were mainly observed in CMM. However, unlike previous studies that demonstrated a higher proportion of *BRAF non-V600E* mutations MMM, our cohort showed that *BRAF non-V600E* mutations were also dominant in CMM. This variation could be attributed to the substantial difference in the proportion of MMM between the two cohorts.

Immunotherapy has emerged as a standard treatment option for melanoma patients due to the limited availability of targetable driver mutations. Previous studies have shown that immunotherapy can provide benefits to melanoma patients, but the prognostic outcomes vary among cohorts with different gene mutations. Douglas et al. demonstrated that the overall response rate (ORR) in melanoma patients (n = 11) with positive *NRAS* mutations was significantly higher (64% vs 30%) than those with wildtype *NRAS* (n = 33) after receiving immunotherapy^30^. However, Michael et al. suggested the similar ORR in both *NRAS* mutant and wildtype melanoma patients^31^. These results indicate that the impact of *NRAS* mutations on the prognosis of immunotherapy still needs further exploration. Furthermore, since CMM was the dominant subtype in both Caucasian cohorts, these results may not reflect the immunotherapy outcomes in Asian cohorts due to ethnic and pathological differences. The Asian study conducted by Zhou et al. included both CMM (n = 92) and non-CMM (n = 114) subtypes in the cohort, demonstrating that the response rates in patients with *NRAS* mutations from both CMM and non-CMM groups were significantly lower than those without NRAS mutations^16^. However, this pooled analysis included three different PD-1 inhibitors (toripalimab, pembrolizumab, SHR-1210), which may have led to biased conclusions. Additionally, the predominant pathological subtype in Asian melanoma patients, ALM, was not further analyzed in the non-CMM group. In our study, we analyzed the immunotherapy outcomes in CMM, ALM, and MMM subtypes after administering only one PD-1 inhibitor (pembrolizumab). In contrast to the above-mentioned study, the response rates showed similar results in both *NRAS* mutant and wildtype groups in CMM. However, patients with *NRAS* wildtype showed a better response than those with *NRAS* mutations in ALM. The variation in prognosis outcomes in CMM between our study and Zhou et al.'s study may attribute to differences in the cohorts. The aforementioned Asian study included melanoma patients from four clinical trials who received different PD-1 inhibitors. The diversity in patient inclusion criteria and complex treatment strategies across different clinical trials could affect the conclusions drawn.

In addition to SNVs, recent studies have discovered that the expression of *ALK* is associated with the efficacy of immunotherapy in melanoma. A previous study demonstrated a prevalence of *ALK* expression of 3% (16/603) in CMM^32^. However, in our study, the rates of ALK expression in CMM and ALM were 41% and 42%, respectively. Such a significant difference can be attributed to variations in the specificity of the screening tools used in both cohorts. The previous study and our study employed immunohistochemistry (IHC) and RNA sequencing (RNAseq) to detect ALK expression, respectively. Previous research has shown that the specificity of IHC is only 52.2%^33^. Furthermore, IHC specificity is highly dependent on the type of antibody used and the cutoff determined by the operators. Therefore, compared to IHC, RNAseq can more accurately reflect the true proportion of ALK expression-positive patients in melanoma. Co-occurrence can be observed between SNVs and *ALK* expression. Previous research demonstrated a high prevalence of *ALK* expression in melanoma patients with wildtype *BRAF* or *NRAS*^34,35^. The same phenomenon was confirmed in our study. Furthermore, studies have found an association between *ALK* expression and prognostic outcomes. A previous study showed that *ALK* expression has a favorable impact on the prognosis of *NRAS*-mutated melanomas^36^. Additionally, the status of *BRAF* and *KRAS* did not have a significant relation to prognostic outcomes in patients with ALK expression. Similar to the previous study, we did not find an association between *ALK* expression and prognosis in the *KRAS* mutant group. On the contrary, *ALK* expression had a favorable impact on the prognosis of *NRAS* wild type melanomas. Furthermore, in the *BRAF* mutant group, better prognostic outcomes were observed in patients with positive *ALK* expression. We speculate that the different results between our study and the previous study are due to differences in the screening tool and cohort size. The aforementioned study employed IHC to detect ALK expression, which may have underestimated the number of patients with ALK expression. Additionally, only 22 patients were included in the cohort, which could have resulted in biased results. In addition to the co-occurrence between SNVs and *ALK* expression, our study also demonstrated that *ALK* expression alone can negatively impact the prognosis of melanoma patients with both CMM and ALM. This suggests that *ALK* expression alone can serve as an indicator to predict immunotherapy efficacy.

Several limitations should be acknowledged in our study. Information on patient characteristics, such as stages, primary or metastatic status, was missing. This information could provide a more comprehensive exploration of the association between genomic mutations and clinicopathological features. Additionally, progression-free survival (PFS) and overall survival (OS) were not evaluated in the study due to the fact that most patients received multiple lines of therapy, which would make PFS and OS less reflective of the effect of SNVs and ALK expression on the prognosis of patients receiving immunotherapy. Furthermore, relatively small sample size for MMM, impacting the generalizability and robustness of the findings. Larger cohort are needed to confirmed the findings in the future. Moreover, future studies should explore the efficacy and outcomes associated with other inhibitors to fully understand the spectrum of responses in melanoma patients.

## Conclusions

In conclusion, our study presented a comprehensive genomic and pathological profiling of Chinese melanoma patients. We identified that *NRAS* and *TERT* mutations have a negative impact on the prognosis of patients with ALM and CMM, respectively. Moreover, we found that *ALK* expression serves as a prognostic indicator in melanoma patients receiving immunotherapy.

### Supplementary Information

Supplementary Figure S1.

## Data Availability

All data used in this study were available in the main text and supplementary.

## Abbreviations

*ICI*: Immune checkpoint inhibitors
*ALM*: Acral lentiginous melanoma
*CMM*: Cutaneous malignant melanoma
*MMM*: Mucosal malignant melanoma
*SNVs*: Single nucleotide variants
*CNVs*: Copy number variants
*SV*: Structural variants
*RFS*: Relapse free survival
*ORR*: Overall response rate
*PFS*: Progression-free survival
*OS*: Overall survival
