# Supplementary material for: Comprehensive insights on genetic alterations and immunotherapy prognosis in Chinese melanoma patients
Source: Sci Rep. 2024 Jul 18;14:16607. doi: 10.1038/s41598-024-65065-6 (PMC11258252; doi:10.1038/s41598-024-65065-6)
Supplement: Supplementary file 1 — Supplementary Figure S1. [file 41598_2024_65065_MOESM1_ESM.docx]

Comprehensive Insights on Genetic Alterations and Immunotherapy Prognosis in Chinese Melanoma Patients

Dong-Dong Jia^1^, Tao Li^1^

^1^ Department of Bone and Soft-tissue Surgery, Zhejiang Cancer Hospital, Hangzhou Institute of Medicine (HIM), Chinese Academy of Sciences, Hangzhou, Zhejiang 310022, China


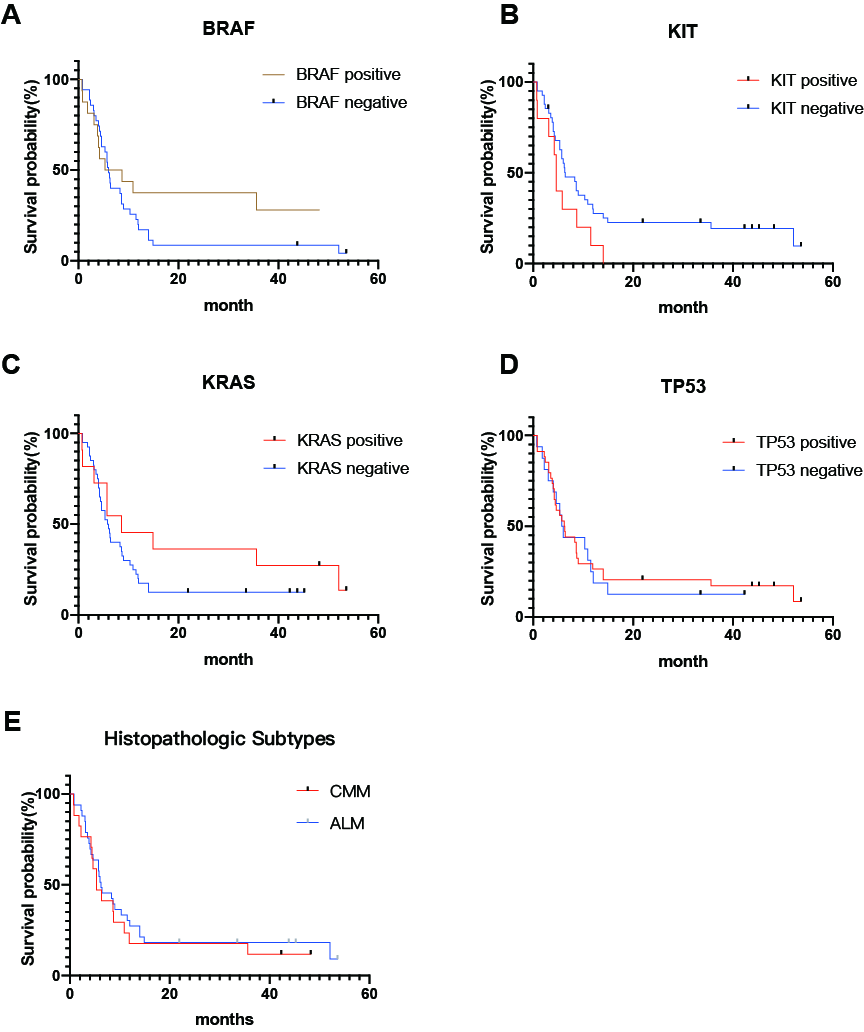


Figure S1. The Kaplan-Meier curves for RFS in patients with SNVs and different pathological subtypes in prognosis after PD-1 therapy in melanoma patients. A, *BRAF* mutations did not show significant effect on the prognostic outcomes in melanoma patients. B, *KIT* mutations did not show significant effect on the prognostic outcomes in melanoma patients. C, *KRAS* mutations did not show significant effect on the prognostic outcomes in melanoma patients. D, *TP53* mutations did not show significant effect on the prognostic outcomes in melanoma patients. E, melanoma pathological subtypes did not show significant effect on the prognostic outcomes in patients (CMM vs ALM). Positive, the specific genetic mutations were detected in patients by DNA sequencing. Negative, the specific genetic mutations were not detected in patients by DNA sequencing. RFS, relapse-free survival.
